# Microfenestrated split thickness skin grafts: an underused technique?

**DOI:** 10.1308/003588412X13373405386015o

**Published:** 2012-09

**Authors:** JET Wokes, A Ali-Khan

**Affiliations:** County Durham and Darlington NHS Foundation Trust,UK

## BACKGROUND

One of the recognised complications of resurfacing a wound with a split skin graft is collection of haematoma or seroma under the graft. This can result in graft loss and delayed wound healing. The risk can be reduced by meshing or fenestrating the graft, which permits fluid escape, but both techniques may compromise the final cosmetic appearance compared with a sheet graft.^1,2^

## TECHNIQUE

**Figure 1:**
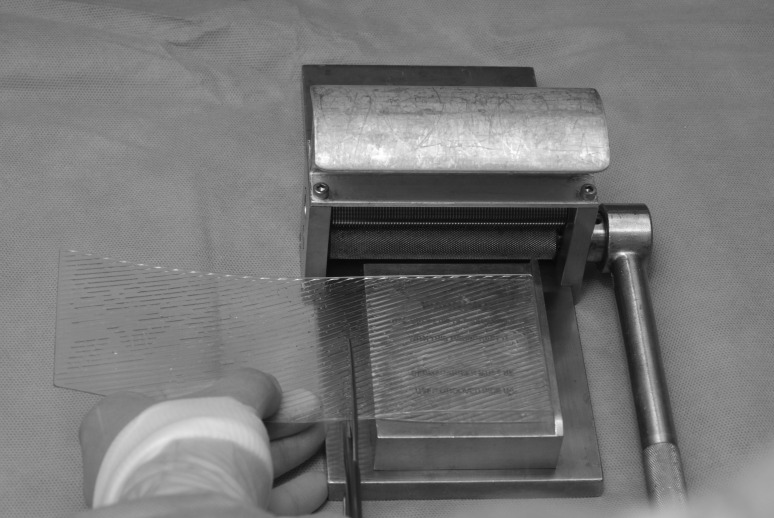
Dermacarrier rotated 90º and cut into thirds

**Figure 2:**
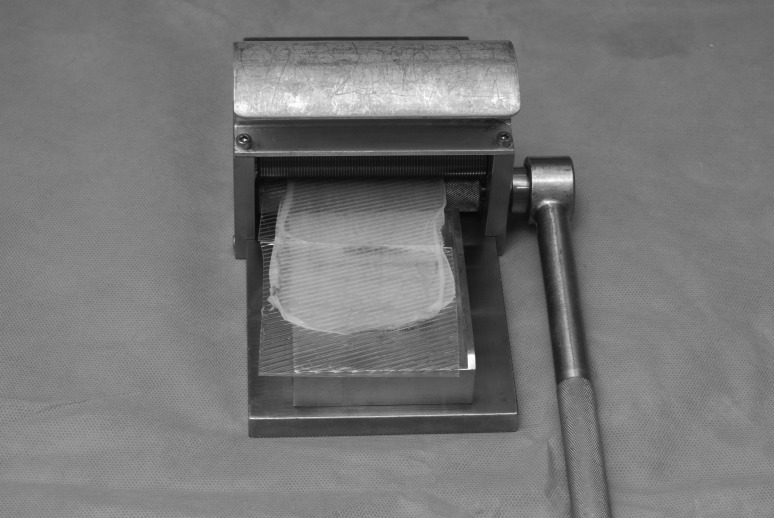
Split skin graft applied to dermacarrier and processed through the mesher

**Figure 3:**
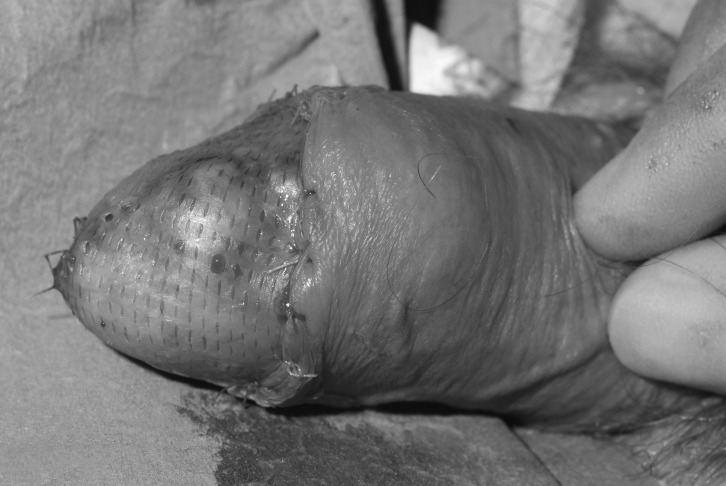
Microfenestrated graft applied to recipient site

**Figure 4:**
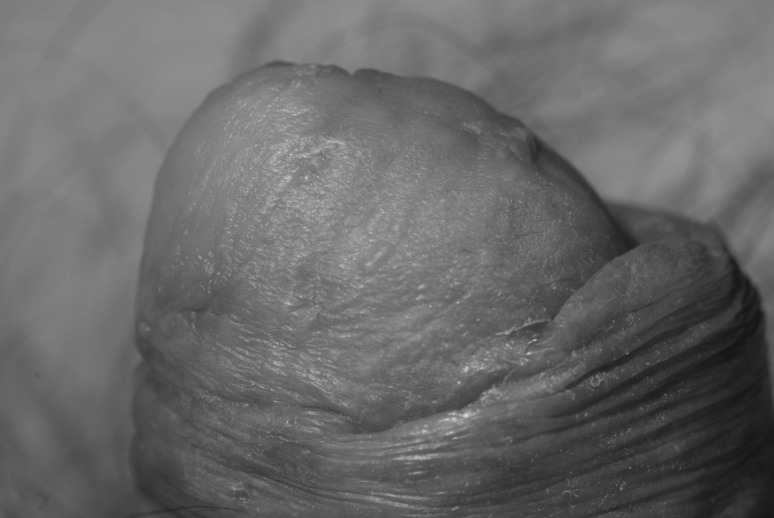
Post-operative outcome

We promote a technique that combines the excellent cosmesis of a sheet graft but with the safety of meshed or fenestrated grafts. First described for the management of burn wounds,^3^ a standard split skin graft is harvested and processed through a Zimmer® skin graft mesher (Zimmer, Swindon, UK) after the dermacarrier has been cut into thirds and rotated 90º (Figs 1 and 2). This results in uniform, small microfenestrations that are easily re-epithelialised, without the cosmetic compromise of meshing and without the risk of tearing or irregularity of fenestrating (Figs 3 and 4).

## DISCUSSION

Though the technique of micromeshing was first described in 1999,^3^ our impression is that this technique is underused and possibly underrecognised. We recommend its routine use whenever a highly vascular wound requires resurfacing without aesthetic compromise.
